# Supplementary material for: Are carbon emissions trading and green financial instruments synergistic? -Comprehensive quantitative research based on content analysis
Source: PLoS One. 2024 Mar 7;19(3):e0298601. doi: 10.1371/journal.pone.0298601 (PMC10919601; doi:10.1371/journal.pone.0298601)
Supplement: S1 Data — (ZIP) [file pone.0298601.s001.zip › Data/地级市Z_F工作报告.zip!/╜¡╦╒/╒≥╜¡╩╨/└┤╫╘╣1⁄2╓┌║┼í╛┬φ┐╦╩2╛▌═°í┐_2013.docx]

各位代表：

　　现在，我代表镇江市人民政府向大会作工作报告，请予审议，并请各位政协委员提出意见。

　　2012年工作回顾

　　刚刚过去的一年极不平凡。在省委、省政府和市委的正确领导下，我们团结和依靠全市人民，紧紧围绕迎接十八大、学习贯彻十八大精神这个大局，全力落实市委的各项决策部署，对标找差争位次、创先争优求作为，较好完成了市六届人大五次会议确定的目标任务，扎实推进了市七届人大一次会议提出的各项工作。镇江的发展在过去的良好基础上，呈现快节奏、展示新气象、取得新成绩。

　　一、全力以赴保增长，经济发展跃上新台阶

　　针对复杂多变的经济形势，在指导思想上，坚持“稳中求进”，加强分析研判，切实把稳增长放在突出位置；在奋斗目标上，确立“质量指标争先、总量指标前移、速度指标进位”的工作定位；在重点措施上，突出企业主体，每月召开一次企业家早餐会，深入企业开展“三解三促”活动，出台十大帮扶措施，及时帮助企业解决实际问题，有力促进了实体经济发展，全市企业亏损面11.5%、为全省最低。建立科学考评体系，以旬保月狠抓推进，促进经济企稳回升。预计全年实现地区生产总值2630亿元，比上年增长12.5%；公共财政预算收入215.5亿元，增长18.5%；固定资产投资1500亿元，增长22%；社会消费品零售总额767亿元，增长15.5%。开放型经济逆势上扬，实际利用外资23亿美元，增长27%；进出口总额116亿美元，增长15%。

　　二、狠抓项目促转型，结构调整迈出新步伐

　　坚持项目化推进现代化的工作要求，集中精力抓项目、调结构、促转型。狠抓重大产业项目。通过每月重点调度、双月现场观摩、季度挂钩督查等办法，不断加压奋进，项目建设速度明显加快，爱励铝业、中冶东方等重大项目竣工投产；丹徒神华基地、京口信威无线产业园等10个超百亿元项目成功落户。累计签约央企项目48个、投资总额1327亿元；签约台资项目49个、投资总额52亿美元。狠抓科技类项目。引进和建设11家重点研发和检测机构，率先在全省实现大中型工业企业研发机构“全覆盖”。高新技术产业产值占规模工业比重居全省第二。荣获首批“国家知识产权示范城市”称号。国家“千人计划”总数突破30人，省“双创”计划超百人，各类高层次人才数量突破2万人。狠抓载体类项目。十里长山高校园区开工建设，镇江生态新城规划编制启动，润州工业园区成为省级高新区并积极争创国家级高新区。镇江新区成为国家级园区循环化改造示范点，中瑞镇江生态产业园正式落户，国家级综合保税区申报工作加快推进。丹阳创成全省首家军民结合产业基地；不再设立化工园区。高资化工园区、句容临港工业集中区加快向循环经济产业园转型。狠抓服务业项目。镇江软件科技产业园奠基，国家数字出版基地镇江园区获批。成立“三山”景区管委会，并创成国家5A级旅游景区，茅山风景区获评全国健康养生示范区，世业洲、茅山湖成为省级旅游度假区。金融机构突破200家，新增社会融资总额650亿元。狠抓农业项目。粮食生产实现“九连增”，新增省级现代农业产业园区1家、亩均效益万元以上设施农业面积12万亩，“三资”投入农业突破60亿元。水利事业加快发展，成为省级水利现代化试点市。

　　三、突出重点快推进，城乡建设展现新面貌

　　根据山水花园城市的总定位，全力破解要素制约，加快推进城乡发展一体化。确立规划龙头地位。完善城乡规划体系，调整市规划委员会，实行规划审查会、首席规划师和镇村规划师制度，主城区按“一体两翼、一核四区”有序整合。狠抓城建重点工程。市区完成投资524亿元，完成房屋征收拆迁255万平方米。扬溧高速西、南出入口整治和体育会展中心等20项城建重点工程顺利竣工，北固山景区修缮等项目建成开放，市档案馆新馆等投入使用，伯先路等历史文化街区彰显特色。首批“7+1”旧城区城中村改造项目启动实施。官塘新城建设全面启动。泰州大桥建成通车。开展环境综合整治。沿江化工企业环境安全专项整治、东部地区与韦岗地区环境整治、秸秆禁烧取得明显成效。历经7年努力，全国第5家通过国家生态市考核验收。顺利通过国家卫生城市复查，成为国家低碳试点城市和省城市管理优秀城市。全面完成省下达的节能减排任务。京沪高铁镇江段沿线环境整治成效明显。“万顷良田”建设、矿山复绿工作走在全国前列。水源地一级保护区实现封闭管理，金山水厂深度处理工程正式供水。统筹城乡发展。“三新”建设成效明显，建成农民安置房380万平方米，新集中安置15600户。累计完成3975个村庄环境整治任务，整治率达57%。

　　四、加大力度惠民生，人民生活得到新改善

　　切实把保障和改善民生作为工作的出发点和落脚点。九大类民生支出155.6亿元，同比增长25.1%，高于公共财政预算支出增幅9.7个百分点。着力提高群众收入。成为全国首批创业先进城市，成功扶持创业1.1万人，城镇登记失业率2.33%。城镇居民人均可支配收入30640元，农民人均纯收入14750元，均实际增长12.3%。切实加强社会保障。社会保障体系建设和社会救助工作走在全省前列。被评为“全国慈善七星级城市”。新建保障性安居工程17979套、拆迁安置房219万平方米，市区逾期一年以上的拆迁安置问题得到有效解决。积极实施惠民工程。推行公交惠民举措，公交票价全省最低。市区建成10个菜市场、5个邻里之家、24家平价商店。文化惠民活动丰富多彩，全民健身活动扎实开展。

　　五、创新管理抓落实，社会建设呈现新局面

　　大力创新社会管理。“网格+网络”社区管理服务模式走在全省前列，城乡和谐社区达标率为75%。文明城市创建年度国家测评列江苏提名城市之首。统筹发展社会事业。国家学前教育体制改革试点取得新突破，5个辖市区通过全国义务教育发展基本均衡县评估。国家公立医院改革试点深入推进，“健康镇江”行动积极实施。统计、供销、人口计生、国家安全、地方志、防震、科普、妇女儿童、残疾人和关心下一代等事业进一步加强；国防教育、人民武装、双拥、人防、民族宗教和援藏援疆等工作取得新成绩。保持社会和谐稳定。落实社会稳定风险评估机制，深入推进“平安镇江”、“法治镇江”建设，积极开展“六五”普法，信访稳定工作走在全省前列。安全生产、食品药品和产品质量安全工作扎实开展。

　　六、尽心尽力履职责，自身建设取得新提升

　　坚持依法行政。自觉接受市人大及其常委会的法律监督、工作监督，主动接受市政协的民主监督，认真听取各民主党派、工商联、无党派人士和人民团体的意见，由政府系统承办的人大代表建议255件，满意率96%；办理政协提案647件，办复率100%，满意和基本满意率100%。法治政府建设实现阶段性目标。大力简政放权。市级审批主体100%、许可审批事项97.7%进行政服务中心，比例均为全省最高。简化招商人员因公出国（境）手续。新增1个国际友好城市。优化公共服务。市政府12345服务热线成为优质服务品牌。政务公开、行政督查、监察与审计监督力度明显加大。事业单位法人治理结构建设试点全国领先。机关事务管理服务得到加强。

　　需要向大会报告的是，对照市六届人大五次会议确定的任务，主城区老铁路范围内的“城中村”改造、梦溪广场改造等项目未能如期实施，主要原因是规划进行了调整。对此，我们及时替换了新项目，保证了投资总额不变、项目个数不减。在今后的工作中，我们将加以防止和克服，确保人代会确定的各项任务顺利完成。

　　各位代表，一年来的生动实践，使我们深深感到：只有坚持“对标找差、创先争优”，才能清醒地认识和把握自己，始终保持加压奋进的紧迫感和责任感；只有坚持“能干事、敢担当、会操作、善创新”，才能焕发出越干越想干、越干越会干、越干越能干的精神风貌；只有坚持“行胜于言”，才能切实增强执行力，说了算、定了干，言必行、行必果；只有坚持“一切为了人民、一切依靠人民”，才能汇聚起建设令人向往的现代化山水花园城市的强大合力！

　　各位代表，过去一年的成绩来之不易。这是在省委、省政府和市委的坚强领导下，全市上上下下、各个方面奋发进取、团结拼搏的结果。在此，我代表市人民政府，向全市人民，向各位人大代表、政协委员、离退休老同志，向市各民主党派、工商联、无党派人士、人民团体和各界人士，向驻镇人民解放军、武警官兵和人民警察，向垂直部门和驻镇单位，以及所有关心支持镇江建设的海内外朋友们，表示衷心的感谢和崇高的敬意！

　　在总结成绩的同时，我们也清醒地认识到，发展中还存在着一些问题：一是经济总量不够大，发展层次还不够高；二是产业布局分散，集中集聚集约程度不高；三是推动经济转型升级的大项目、好项目还不够多，招引项目的力度还需加大；四是城乡规划建设管理还需进一步加强，城市形象和对外影响力有待进一步提升；五是政府的创新能力、执行能力、服务意识需要继续增强，投资环境需要进一步改善。对这些问题，我们将高度重视、认真解决，以新的作为、新的变化，不辜负各位代表和全市人民的期望！

　　2013年主要工作

　　2013年是全面贯彻落实十八大精神的开局之年，是实施“十二五”规划承前启后的关键一年，也是我市率先基本实现现代化的重要一年。从国内外形势看，尽管世界经济进入深度调整期，错综复杂、充满变数，低速增长态势仍将延续，但我国仍处于重要战略机遇期，发展趋势总体向好，经济增长的动力和活力将进一步增强；从我市看，尽管各种压力和挑战仍在，但更面临诸多重大机遇。只要我们坚持“创新争先、实干兴市”，就一定能圆满完成各项目标任务，开创镇江现代化建设的新局面！

　　今年政府工作的总体要求是：深入贯彻党的十八大精神，按照中央、全省经济工作会议和市委六届四次、五次全会部署，紧紧围绕科学发展主题、转变发展方式主线，以提高经济增长质量和效益为中心，深化对标找差，奋力创新争先，大兴实干之风，扎实推进“八项工程”，深入实施产业升级、城乡建设、创新创业、民生改善“四大行动计划”，全力建设令人向往的现代化山水花园城市，全面加快率先基本实现现代化进程。

　　全市经济社会发展的主要预期目标是：地区生产总值增长11.5%；公共财政预算收入增长18%；固定资产投资增长21%；社会消费品零售总额增长15%；实际利用外资增长15%；进出口总额增长8%；研发经费支出占GDP比重2.45%；城镇居民人均可支配收入实际增长11%；农民人均纯收入实际增长12%；城镇登记失业率控制在3.5%以内。全面完成省下达的节能减排约束性指标。

　　工作中，我们将牢牢把握稳中求进这一基调，全力推动经济企稳回升，确保在全省质量指标“保五争先”、速度指标“保六争先”、总量指标位次前移；紧紧抓住转型发展这一关键，抓抢重大发展机遇，不断优化空间布局，加快构建现代产业体系，大力推进生态文明建设；始终践行以人为本这一理念，不断保障和改善民生，便民利民安民富民，着力提高人民的幸福程度；全面体现提升水平这一要求，高标准谋划发展，高质量推进工作，高效率开展服务。

　　2013年的任务艰巨而繁重，我们将按照十八大“五位一体”的总体布局，紧紧围绕市委六届五次全会提出的“四大行动计划”，全力抓好以下八个方面：

　　一、推动产业集中集聚集约发展，促进经济转型升级

　　加快建设先进制造业特色园区。重点围绕高端装备、新材料、新能源、航空航天、新一代信息技术、生物技术与新医药六大千亿级战略性新兴产业，规划建设20个先进制造业特色园区。丹阳重点规划建设高端装备制造、新材料、航空航天、生物技术与新医药等特色园区，积极推进眼镜、汽车及零部件、五金工具等特色产业集群发展；句容重点规划建设新材料、新一代信息技术等特色园区；扬中重点规划建设智能电器、新能源等特色园区，积极推进工程电气等特色产业集群发展；丹徒重点规划建设高端装备制造、新能源等特色园区，积极推进轴承、绿色食品加工等特色产业集群发展；京口重点规划建设航空航天、新一代信息技术等特色园区；润州重点规划建设船舶及海洋工程、海水淡化及水处理装备等特色园区；镇江新区重点规划建设新材料、新能源、航空航天、生物技术与新医药等特色园区。推进工业化与信息化深度融合，加快改造提升传统产业。

　　加快建设现代服务业集聚区。重点打造30个集聚区。现代旅游业：高起点编制全市旅游产业发展总体规划，旅游项目总投资超300亿元。实施“三山”景区综合提升项目、世业洲省级旅游度假区、佛祖舍利文化园等“十大工程”，实现旅游综合收入630亿元。现代物流业：依托沿江、沿河与沿路等交通节点优势，规划布局物流产业园区，实现营业收入500亿元。文化产业：加快建设国家数字出版基地镇江园区、十里长山文化产业园等重点文化产业集聚区，实现营业收入450亿元。现代商贸业：依托主城核心区、大港、丁卯、丹徒新城和南徐新城，全力打造“一主四副”五大商贸中心，培育交易额超百亿元的市场2家。各辖市都将培育高端商贸集聚区。现代金融业：启动长江路商务金融集聚区建设，引进2—3家股份制银行。信息及科技服务业：加快镇江科技新城、镇江软件科技产业园与京口大禹山创意产业新社区等一批载体建设，新增软件企业100家以上，营业收入达到400亿元以上。加快发展建筑业，总产值力争完成600亿元。

　　加快建设现代农业产业园区。着力打造30个园区，重点发展优质粮油、应时鲜果、特种养殖、花卉苗木、休闲观光五大优势特色农业。全面启动国家现代农业示范区创建工作，提升省、市两级现代农业产业园区和省级农产品加工集中区的建设水平，新增农业园区核心区面积5万亩，高效设施农业比重达16%以上。实施农业产业千亿工程，新增省级农业产业化龙头企业5家、农业适度规模经营10万亩。加快城乡水利建设，总体达到基本现代化标准。

　　加大项目建设和企业培育力度。坚持和完善每月调度、双月观摩、季度督查、年度考核等机制，全力推进200个、总投资3545亿元的市级重点产业项目。按照“一个重大项目、一名牵头领导、一套专门班子、一个具体方案、一抓到底”的要求，加快推进重大招商项目。全力帮助企业做大做强，力争新增百亿企业1—2家；特别关注和扶持中小企业、小微企业发展。加快光伏等产品的推广应用，帮助企业渡过难关。大力实施品牌战略，增强企业核心竞争力。

　　二、全力拼抢三大机遇，增强区域综合竞争力

　　抢抓苏南现代化建设示范区的机遇。按照“现代产业集聚、科技创意人才汇聚、城乡统筹发展、生活品质优越的生态文明先行区”的战略定位，建立“资本+政府+产业”发展新模式，继续深化镇江生态新城的规划编制工作，适时启动一期“茶花岛”建设。各辖市区都将找准努力方向，明确工作重点，抓住这一历史性机遇，迈出区域转型发展的新步伐。

　　抢抓宁镇扬同城化的机遇。积极推进基础设施体系建设，规划镇江至禄口机场高速公路、连接三市中心城区的轨道交通主干线，抓好312国道南移、镇江至丹阳、句容和扬中的快速通道以及镇江火车站南北一体化改造、镇荣公路丹徒段改造、沪宁高速支线改造、苏南运河“四改三”、大路通用机场升级、大港四期工程等建设。积极推进区域创新体系建设，利用宁镇扬高校和科研院所资源，在项目建设和产学研合作上获取共赢利益。积极推进共同市场体系建设，着力消除市场分隔，构建无障碍、同城化的统一大市场。

　　抢抓国家推进新型城镇化的机遇。把城镇化作为扩大内需的最大潜力，启动新一轮“三新”建设，坚定不移地走集约、智能、绿色、低碳的新型城镇化道路，积极争取国家新型城镇化试点城市。有序推进农业转移人口市民化，城镇化率达到65%。中心城区加快南拓步伐，丹徒加速与主城融合；丹阳启动练湖水城建设，加快与镇江市区对接；句容全力打造“五点一线”，加快与南京的全面融合；扬中以举办省第八届园艺博览会为契机，推动城市品质的全面提升。

　　三、坚持创新驱动，全面加快科技创新步伐

　　建设科技创新载体。创成国家级高新技术产业开发区，建设镇江知识城、3个全球研发中心、3个央企研究院和3个院士工作站，全力推进中船船用设备与海工配套产业园建设。积极推动丹阳和扬中争创省级高新区。镇江科技新城探索建立专利产业化和专利营运集聚实验区。加快丹阳军民结合产业基地、句容宝华创新创业基地、扬中智能电网产业岛等载体建设。

　　突出企业主体地位。狠抓科技创新100个重点项目，新增高新技术企业100家。巩固大中型工业企业研发机构全覆盖成果，确保省级以上研发机构比重提高5个百分点以上。充分发挥高校与科研院所的作用，与企业共建联合创新平台。成立市技术产权交易所、镇江·以色列高新技术交易中心，建设省级科技金融合作创新示范区。切实加强知识产权运用与保护。

　　打造“人才特区”。出台苏南最具竞争力的政策，营造一流的工作生活环境。实施创新创业领军人才集聚计划，引进100个创新创业领军人才团队，国家“千人计划”、省“双创”计划人数继续走在全省前列。积极实施“百千万”人才计划，培养提升100名以上创业企业家，培养引进1000名以上企业经营管理人才和1万名高技能人才。

　　四、迎难而上求突破，大力推进山水花园城市建设

　　强化规划引领。抓住城市总体规划修改的契机，全面提升规划的现代化、国际化水平，完善多中心、组团式、网络型城市空间结构。加快规划审批流程。按照“特色鲜明、品质高雅”的要求，推进重点区块、重要节点城市设计。

　　加大建设力度。重点实施285个城建项目，全年完成投资900亿元以上，竣工重点项目40项。启动市域快速路网建设，官塘桥路建成通车，开工建设五凤口高架。新建、改建市区主次干道48条，实施中山西路“四改六”工程，打通健康路、大西路、学府路三期等5条断头路、瓶颈路。“7+1”旧城区城中村改造项目完成征收拆迁并开工建设，启动铁瓮城棚户区等20个片区、孟家湾等13个“城中村”改造。结合市政府旧址改造，建设时尚文化街和市民活动中心。开工建设中山东路、电力路地下人防工程。完善官塘新城基础设施，加快推进招商南山创意园项目，建设科技馆。十里长山高校园区完成管线迁移，建设部分道路和配套设施，市委党校、省交通技师学院、镇江高专教学楼主体封顶，镇江技师学院投入使用。

　　加强精细管理。强化区级管理职能，有效调动街道、社区的积极性。全面提升“数字城管”水平。开展环卫设施达标行动，建设灰渣处置场和餐厨废弃物处置中心，城市生活垃圾无害化处理率达100%。出新长江路、南门大街沿街立面，完成京沪高铁、宁杭高铁、润扬大桥、泰州大桥出口及沿线环境整治，建成4条市容管理示范路。继续开展专项整治，有效解决违章占道、渣土扬尘等突出问题。加强对渣土车、电动车与人力三轮车的源头管控。狠刹违建之风，对建成区存量违建三年内全部拆除，对新建违章做到发现一起拆除一起，一律不予补偿。实施2980个村庄环境整治，全面完成整治任务，并建立长效管理机制。

　　五、充分彰显独特优势，高标准推进生态文明建设

　　建设低碳示范城市。扎实推进国家低碳试点城市建设，深入实施好“九大行动”。实施主体功能区规划，明确优化开发、重点开发、限制开发、禁止开发区域，划定生态“红线”，将47个重要生态功能保护区落到实处，确保生态红线区面积高于20%；加强碳汇建设，编制碳排放清单，探索建立碳排放交易市场；全面实施镇江经济开发区国家级园区循环化改造，建设中瑞镇江生态产业园；建成南山10公里绿道并实现贯通；投放1000辆以上公共自行车，推进智能、低碳公交和运输，倡导低碳生活方式。

　　继续加强生态建设。巩固国家生态市创建成果，实施生态文明建设规划，镇江和丹阳、句容、扬中、丹徒力争全部进入全国生态文明建设试点城市行列。加大生态修复力度，加快滩涂、湿地、丘陵、山体和工矿废弃地恢复治理，积极开展土壤污染防治和修复。新增造林面积4万亩，新建小区绿化率达到35%以上，力争创成国家森林城市。

　　推进环境专项治理。东部地区开展挥发性有机物专项治理，完成索普化工基地、镇江新区化工园区周边隔离带拆迁工作。高资化工园区创成省级循环经济产业园。加大韦岗地区环境整治力度，取缔关闭非法轧石企业和石灰窑。深入开展第三轮化工行业整治，关闭企业50家以上，化工园区产业集中度达到65%。推进重金属行业整治。加强水环境综合治理，提高地表水功能区达标率。对凌塘水库实施生态修复，建设大港水厂，改建征润洲取水口，实施离岸取水工程，确保安全供水。

　　六、全面深化改革开放，不断增强发展活力

　　推进体制机制创新。深化行政审批制度改革，扎实推进经济发达镇改革试点和事业单位分类改革。积极开展户籍管理制度改革，充分吸纳外来人口。有效落实“营改增”试点政策，全面推行预算绩效管理。稳妥推进市级投融资平台改革，不断增强其产业发展能力。引进战略投资者，推动恒顺、索普等国有企业改革。认真落实各项政策，大力支持民营经济发展。加快构建新型农业经营体系，积极探索农村金融、土地制度改革，深入推进丹阳农村改革试验区建设。

　　增强招商选资实效。突出产业招商、要素招商、以商引商和小分队招商，力争引进5亿美元以上项目10个、10亿美元以上项目3个，全年实际利用外资冲刺30亿美元，吸引央企投资100亿元、市外民资360亿元以上，各辖市区分别引进一个总投资100亿元以上的产业项目。举办金秋经贸文化旅游洽谈会以及赴台招商、国际友好交流、华创会等活动。创成国家级综合保税区，全力支持丹阳争创国家级经济技术开发区。

　　积极开拓外需市场。巩固欧美日等传统市场，开拓东盟、印度、俄罗斯等新兴市场，通过以新补旧，推动外贸稳定发展。加快培育重点出口企业集群，提高品牌产品出口比重，力争全年境外注册商标超过100个。组织企业参加各类展会，鼓励企业“走出去”，大力提升服务外包水平。

　　七、保障和改善民生，着力提高人民的幸福程度

　　增加居民收入。实施收入倍增计划，广辟增收渠道，突出农民、企业职工、中低收入者和困难家庭“四个群体”增收，提高中等收入者比重。实施就业优先战略，扶持创业7000人，带动就业3.5万人；新增城镇就业6.5万人、转移农村劳动力2万人。提高企业最低工资标准和工资指导线，深化职工工资集体协商制度。

　　强化社会保障。统筹推进城乡社会保障体系建设，完善工伤、生育保险市级统筹和失业保险市区统筹，力争覆盖率98%以上。完善机关事业单位养老保险制度，推动被征地农民进“城保”，实现医疗保险异地就医联网结算。开辟外来务工人员和引进人才等社保绿色通道。实现城乡低保标准一体化。新建保障性住房8800套、拆迁安置房293万平方米。切实解决中等偏下收入家庭住房困难问题。努力保持价格总水平基本稳定。加强养老服务体系建设。切实保障妇女、儿童、残疾人和社会弱势群体的合法权益。积极发展慈善事业，增强社会救助能力。

　　建设“四公两场”。扎实推进公园、公交、公厕、公墓和农贸市场、停车场建设。完成黄鹤山、凤凰山等8座山体公园式改造，实行免费开放。贯通公交专用通道，新增空调车、新能源公交车200辆以上，基本建成智能公交系统，增加开行班次，着力提高准点率。主城区“10分钟公厕服务圈”覆盖率达到70%。建设公益性骨灰安息堂和平价公墓。市区新建9个邻里中心、4个菜市场，升级改造2个菜市场，新增平价商店14家；新增停车场泊位3000个，建成道路停车信息化管理系统，实行梯级收费。

　　八、大力发展社会事业，推进社会管理创新

　　创建文明城市。以争创全国文明城市为龙头，深入开展群众性精神文明创建活动，积极培育和践行社会主义核心价值观，加强未成年人思想道德建设，不断提升市民素质，打造“大爱镇江”等特色品牌。建立城市荣誉制度，表彰基层各类突出贡献者，大力营造褒奖先进、崇尚奉献、弘扬正气的社会风尚。广泛开展志愿者、体验日等主题活动，增强市民的家园意识、公民意识、荣誉意识，形成共建美好家园的生动局面。

　　优化教育卫生服务。加快推进教育现代化，以办人民满意的教育为目标，加强师德建设，提高教学质量。继续深化国家学前教育体制改革试点，经济困难家庭幼儿免费入园，积极推进义务教育优质均衡发展、高中教育优质创新发展，大力支持在镇高校发展。继续推进校舍安全工程，新开工23万平方米。深入推进医药卫生体制改革和“健康镇江”行动，健全区域健康服务体系。加强人口计生综合管理与服务。

　　扎实推进文体惠民。举办“长江音乐节”、“欢乐家园”等系列文化惠民活动，规划建设各类特色文化广场、图书馆、美术馆和文化站室，开工建设西津音乐厅，创建省公共文化服务体系示范区。依法保护历史文化遗产。广泛开展群众体育活动，举办市第十四届运动会，体育会展中心正式运营。

　　加强社会管理。巩固提升“网格+网络”社区管理服务体系，针对性地研究解决人户分离、空挂户问题，城乡和谐社区建设达标率83%以上。切实加强社区物业管理。深入开展劳动关系和谐企业创建活动。认真组织好第五届居委会和第十届村委会换届选举。深入开展全民国防教育，扎实做好人民武装、民兵预备役和双拥工作。深入推进“平安镇江”、“法治镇江”建设，广泛开展“六五”普法教育，依法及时化解各类信访矛盾。严格落实安全生产责任制，加强食品药品安全监管，提高应对和处置突发事件的能力。

　　切实加强政府自身建设

　　全面完成今年的目标任务，向313万镇江人民交上一份满意答卷，需要政府全体公务人员牢记责任、提升能力，夙夜在公、勤勉工作。

　　全面提升执行力。空谈误事，实干兴市。现在“四大行动计划”已制订，目标任务已明确，关键是干，不议论、埋头干，不推诿、我来干，不指责、共同干；关键是落实，明确职责抓落实，传递压力抓落实，严格考核抓落实，把任务变成具体项目，把项目变成具体行动；关键是执行力，加强制度建设，强化首问负责，有令必行、有禁必止；既有部署，更有检查；既看过程，更看结果。

　　全面提升服务能力。坚持每月一次集体学习，提高专业知识水平和实际操作能力。深入开展“三解三促”活动，为群众和企业排忧解难。切实精文简会，少发文、发短文、发有效的文；少开会、开短会、开管用的会。精简并规范各类检查考核，切实减轻基层负担。建立规模以上企业纪检监督定点制度，禁止向企业乱检查、乱收费、乱罚款。

　　全面提升公信力。坚持依法行政，深化法治政府建设，自觉接受市人大及其常委会的法律监督、工作监督和市政协的民主监督，充分发挥各民主党派、工商联、无党派人士和人民团体的参政议政作用，注重发挥新闻舆论监督和社会公众监督作用。及时倾听民声，多渠道、经常性听取各方面的意见建议，不断提高行政决策的民主化、科学化与规范化水平。坚持每月一次政府新闻发布，构建市政府12345服务“热线云”，完善网络发言人制度。强化诚信意识，严格兑现对人民的承诺，说到做到；大力推进社会诚信体系建设。

　　全面提升拒腐防变能力。深入贯彻中央政治局八项规定和省委、市委的十项规定，进一步改进作风，密切联系群众。全面落实党风廉政建设责任制，加快推进惩防体系建设，继续推进行政权力网上公开透明运行，建设风清气正、高效廉洁、人民满意的公务员队伍。

　　各位代表，人民政府为了人民，人民政府依靠人民。让我们在市委的坚强领导下，团结和带领全市人民，解放思想、攻坚克难，齐心协力、扎实工作，把我们的家园建设成为令人向往的山水花园城市，为镇江率先基本实现现代化而努力奋斗！
